# Beyond the flow rate: the importance of thermal range, flow intensity, and distribution for water-efficient showers

**DOI:** 10.1007/s11356-019-07235-y

**Published:** 2019-12-30

**Authors:** Kemi Adeyeye, Kaiming She, Inês Meireles

**Affiliations:** 1 Department of Architecture and Civil Engineering, University of Bath, Bath, UK; 2 School of Environment and Technology, University of Brighton, Brighton, UK; 3 RISCO, Department of Civil Engineering, University of Aveiro, Campus Universitario de Santiago, 3810-193 Aveiro, Portugal

**Keywords:** Flow distribution, Showerheads, Temperature, Water efficiency, Water labelling, Water user satisfaction

## Abstract

Studies show that user behaviours have not necessarily changed, despite the prevalence of water-efficient products in the market. One reason is because the technical emphasis for delivering the water use efficiency of products has focused on reducing the flow rate. Therefore, this study was undertaken to examine the physical parameters that define the technical efficiency of showerheads against the experiential performance (and therefore the satisfaction with the showerheads). These parameters were measured in a controlled laboratory environment and the findings were triangulated against user feedback from in-home trials. Synergies between the laboratory data and user feedback were found. Notably, it was found that water spray intensity, distribution, and temperature loss all impact the quality of showering experience. These factors also influence shower duration—and thus the volume of water used in the shower. Significantly, these technical metrics affected the overall experiential performance of such products from the users’ perspective. Therefore, the design of water-efficient showerheads, in addition to delivering water discharge savings, should avoid poor spray distribution, intensity, and heat retention. The implications of the findings are that water efficiency labelling and product standards should extend beyond the emphasis on limiting the flow rates—typically to 9 l per min for showerheads. This study shows good merit for including the spray intensity (pressure), distribution, and degree of heat loss, in addition to the discharge rate, as part of the performance and efficiency considerations of showerheads.

## Introduction

Water efficiency, as opposed to water conservation, is the optimised use of water commensurate to need. This implies that water must be optimally used and waste eliminated through behaviour, technology, and infrastructure efficiency (Adeyeye 2011; Balnave and Adeyeye 2013). This paper focuses on the technical and not ‘allocative’ efficiency of water (Global Water Partnership (GWP) Technical Committee, 2004). Technical efficiency comprises user efficiency, water recycling, and reuse as well as supply efficiency. Reductions in water consumption can defer investments in the expansion and upgrade of water supply infrastructure as well as the reduction of average and peak effluent loading to the wastewater system (Vieira et al. 2017). It can also lead to energy savings and thus carbon emission’s reduction (Wong et al. 2016; Binks et al. 2016; Dieu-Hang et al. 2017). The adoption, installation, and appropriate use of water-efficient devices are seen as an effective means to promote water use efficiency for the following reasons (Millock and Nauges 2010):

Water consumed through both indoor and outdoor appliances e.g., showers, toilets, washing machines, sprinklers, represent a significant share of households’ daily water use particularly in developed countries.

The reduction potential of water-saving fixtures is now well-acknowledged: among other examples, a standard showerhead may use up to 25 l of water per min whereas a water-efficient showerhead might use as little as 7 l per min.

Strategies to promote installation of water-efficient devices are likely to be more politically acceptable than price increases or policies imposing water restrictions.

The pervasive role of habits in human behaviour may make other forms of non-price policies, such as public information campaigns, yield little effect.

The benefits of eco- or efficient technologies and products such as the use of water efficiency products and fixtures have been found to result in up to 35% of indoor water savings (Lee and Tansel 2013). Several studies (e.g., Jorgensen et al. 2009; Linkola et al*.*^2013^; Beal et al. 2013) have highlighted the importance of pro-environmental water use behaviours for promoting water use efficiency. Others (e.g., Inman and Jeffery 2006) have highlighted the merits of demand-site policy tools such as labelling and metering.

Labelling, when combined with household awareness, understanding, and trust of the labels have been shown to positively affect consumer decisions to adopt water-efficient devices and be energy-efficient (Dieu-Hang et al. 2017). It offers opportunities for increased sales through product differentiation, increased accountability, or increased choice for consumers in a greening retail environment (Horne 2009). But the criticisms are that (Horne 2009):

- There are too many products, too much information, too little time, and a paucity of independent, accessible, readily accessible, and understandable information about environmental performance.
- There is no consensus as to where and to what extent labelling results in sustainable consumption or environmental impacts.
- Consumers are attracted to simple eco-labels because they provide for clear decision making, but simplicity can undermine efficacy of environmental claims.
- Criteria consistency and difficulty of making direct functional comparisons between products can operate against simplicity aims.

Water labels utilize a singular metric—flow rate—as the measure of efficiency. However, studies have found that user perception of product performance can affect the degree of water use efficiency and performance extends beyond the singular issue of flow rates. For example, the water efficiency trials conducted by Lee and Tansel (2013) found that satisfaction pertaining to the product or experience of the use of high efficiency products was closely correlated with the achieved water savings. If low-flow devices do not deliver equivalent service, then customer satisfaction may lead to circumvention of regulations or changes in usage patterns i.e., longer showers(Koomey et al. 1995). The Australian Water Efficiency Labelling Scheme (WELS)-rated showerhead trials in Wong et al. (2016) confirmed this and found that 22 of 37 participants were not satisfied with the flow rate and pressure and thus preferred to use their original showerheads. Among the 15 participants who were satisfied, eight still preferred to use their original showerheads for a more comfortable showering experience while seven would keep using the WELS showerhead. They observed correlations between hot water temperature, temperature difference between hot and cold-water supply, water consumption, and flow rate. Still, they found that full implementation of WELS-rated showerheads with resistance factor *k* ≥ 4.02 can reduce water consumption by 37%, energy use by 25%, and CO_2_ emissions by 26%, showing that the use of a WELS Grade 1 water-efficient showerhead can save water and energy while reducing carbon emissions.

Factor analysis by Bhandari and Grant (2007) found that water sufficiency, reliability of water supply, convenient water-point location, water quality, and water pressure were prime indicators of the users’ degree of satisfaction. Zadeh et al. (2014) found the possibility that users will opt for the higher flush setting or flushing dual-flush toilets multiple times due to a misconception of malfunctioning or poor performance of this type of toilet. Okamoto et al. (2015a,b) studied quantitative showerhead factors as influences on satisfaction and found that the spray force, spray force-per-hole, spray pattern, water volume ratio in spray patterns within φ100 and φ150, temperature drop, and spray angle all influenced satisfaction. They concluded that satisfaction and usage water flow have a spurious correlation relationship. Instead, they proposed that the physical properties of spray force-per-hole and temperature drop, and designs that set an appropriate value for water distribution and spray angle for spray patterns within φ100 and φ150, improves satisfaction independent of usage water flow (Okamoto et al. 2015a). Okamoto et al. (2015b) also found low flow, low satisfaction groups in their comparative study of users in Taiwan, Japan, and Vietnam. Alkhaddar et al.’s (2007) determined that the design of the showerhead itself, flow distribution, skin pressure, and the flow rate are important characteristics of a good shower. They stated that the showerhead design could have a major effect on how the flow distribution changes with the flow rate. Thus, it may be detrimental to restrict the flow in an existing domestic shower without some consideration of the showerhead itself. They also found a very considerable change in skin pressure for a comparatively small change in the flow rate—because skin pressure is related in a non-linear manner to flow rate. This may make it difficult to maintain user satisfaction while reducing the flow of skin pressure if people care about this variable. Therefore, to improve the showering experience, showerheads need to be redesigned by considering the head factor where there is low flow or through technological changes, such as enforced droplet formation by sonic agitation (Alkhaddar et al. 2007).

These studies demonstrate that flow rates alone present an incomplete picture as an indicator of the water efficiency of products such as showerheads. They confirm that water use efficiency requires the renewed understanding of the relationships between the end use and the end users of residential water (Beal et al. 2013), or technological solutions could encourage inefficient behaviours (Linkola et al. 2013).

Knowledge gaps remain about the determinants of household energy and water consumption. First, previous studies rarely bridge the social and technical domains. Thus, Zadeh et al. (2014) advocate a combined approach, achieving 31.9 l per person per day when the most efficient technology options are combined with positive user behaviours. Secondly are gaps on the comprehensive understanding of how individual characteristics, as well as energy and water-efficiency labelling schemes, affect conservation behaviours as well as the factors that affect household decisions about the adoption of energy- and water-efficient appliances (Dieu-Hang et al. 2017). Therefore, this research addresses these gaps by defining, in addition to flow rate, the other important efficiency and performance indicators for water-efficient products. It evaluates the technical efficacy of water-efficient showerheads as a determinant for its socio-behavioural impact, i.e., efficiency in-use. To achieve this aim, laboratory studies are conducted. The findings are then correlated with user feedback provided during in-home trials of the same showerheads. Studies such as this contribute to the better understanding of sustainability mismatch in water-efficient products against user preferences and user behaviours.

## Research context

This study focuses on showerheads because studies have shown that they contribute up to a third of domestic water use and the associated energy use. It builds on work presented in Adeyeye et al. (2017) which demonstrated that the environmental performance of resource using products such as showerheads are affected by product design and use. The study also found that the uptake of a product depends on how a product satisfies user performance expectations in addition to meeting environmental benchmarks and standards. Therefore, water-efficient product design and performance must be studied based on individual products in conjunction with user preferences to determine product’s water efficiency and marketability.

The Market Transformation Programme (MTP 2011) report investigated three shower water consumption scenarios: reference, policy, and earliest best practice scenarios for England and Wales and found that water consumption increases in all three and up to 512,972 Ml/year by 2030 in the worst-case scenario. They projected that 2.6 million showers will be sold per annum in England and Wales by 2030. Therefore, water-efficient showerheads, apart from low-volume WCs and at baseline supply and price levels, are cost effective compared with other fittings (Gleick 2003). A wide range of showerheads with multiple functionalities and characteristics, all designed to meet user needs, preferences, and demands, are now readily available. The question is whether the increasing complexity and the resulting positive or negative perception inform the shower duration, and therefore water consumption during use. Eco-showerheads have different types and characteristic and offer different flow rates/patterns, pressure, temperature ranges (Table 6 in the Appendix; Table 1).

**Table 1.** Summary of showerhead classification (Biermayer, 2005)

Classification	Characteristics
Single head	One or more settings; more and less focused sprays and a pulsating spray.
Multiple head	Two or more spray nozzles connected to one pipe; easily used to replace a single head fixture
Cascading showerhead	Mounted overhead with the water dropping straight down; relatively softer spray and large diameters (6 to 8 inches)
Shower panel or shower tower	Designed to spray water from more than one location with more than one showerhead operating sequentially
Rain systems	Designed to simulate rain by allowing water to fall from an overhead fixture
Body spas	Consisting of multiple showerheads described by some as the vertical equivalent of a spa; showerheads activated sequentially or intermittently

Biermayer (2005) postulated that water consumption could be reduced in several ways depending on information available in relation to the water efficiency and performance of showerheads. However, full knowledge about individual showerheads and an understanding of customer behaviour with respect to showerhead selection and showering practice is needed. This knowledge and understanding can only be attained through research and controlled laboratory testing to:

- Identify and remove from the market those showerheads that exceed the standard for water flow;
- Highlight and promote showerheads that use even less than the standard while providing a good shower experience;
- To encourage replacement of non-compliant showerheads with effective low-flow showerheads by providing consumers with information about which showerheads they are most likely to find satisfactory;
- Identify low-flow showerheads that provide an adequate showering experience in place of a multi-head shower fixture;
- Establish means that would encourage consumers to turn off the water while lathering thus saving water;
- Address perceived or real safety concerns that may prevent utilities from promoting very low-flow showerheads.

## Methods

The methodological design of this particular study is informed by the work of McClelland (2005) and Alkhaddar et al. (2007). The former used focus groups to address the issue of a ‘good shower’. They found that the main requirements were temperature stability, adequate water volume and distribution, and perceived skin pressure. Alkhaddar et al.’s (2007) study proposed measurement methods and the relative dependencies of performance metrics such as flow rate and skin pressure. Adeyeye et al. (2017) present the underpinning user findings and further rationalised the purpose and objectives of this work. Based on these, a mixed methodology approach was utilised which combines objective measurements from laboratory studies and users, with findings from in-use/usability tests and feedback. Laboratory experiments are generally not burdened with the problems that arise in field experiments as the conditions for the experiment can be controlled. However, they do not adequately simulate the context and may lead to less valid data, where there is a potential disconnect between stated preferences, intentions, and actual experiences (Sun and May 2013). Therefore, this combined approach is highly beneficial for improving the validity and reliability of the research findings.

The 10 sampled showerheads are summarised in Table 6 in the Appendix. All the showerheads were water-efficient, discharging water at between 5 and 11 l per min. The regulated flow rate is as supplied by the manufacturer, whilst the measured regulated flow rate is as measured in the laboratory. Therefore, the differences in the measured flow rate is highly dependent on the set-up, pressure differentials, and other situational factors.

**Fig. 1:**
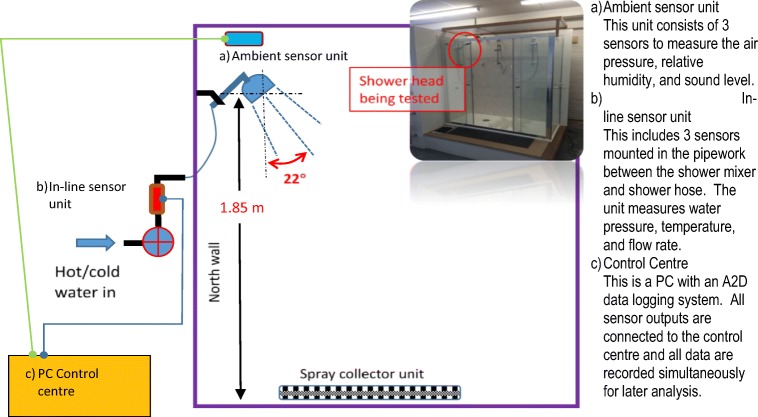
The shower cubicle and instrumentation layout

The laboratory experimental setting and the schematic configuration of the instrument setup in the shower cubicle are shown in Fig. 1. The cubicle has provision for three showerheads to be mounted at the same time. The mounting position for this study is as indicated and the mounting wall is referred to as the north wall for the purpose of orientation.

The laboratory-determined performance of the 10 water-efficient showerheads was considered in conjunction with user feedback. Twelve volunteers participated in a 12-week study where they were able to use each showerhead at home for 1 week. They completed a feedback sheet for each showerhead after their first and last use during the trial week. Therefore, it was possible to compare their initial and final feedback. This enabled deduction of the important showerhead parameters that informed the user perception and feedback of the product.

### Showerhead spray diffusion/distribution

The showerhead spray diffusion and distribution measurement approach revised the method employed by Alkhaddar et al. (2007). Their collector system consisted of several radial zones with each zone collecting water, measured via individual outlet pipes. The design is simple and efficient to derive a distribution map of the spray. However, the fundamental issue with this design is that it assumes that the spray has a circular pattern and is fully symmetrical with respect to the centre of the spray. In fact, the spray pattern is generally not symmetrical in the radial direction for several reasons. Firstly, the spray holes may not be positioned symmetrically, even if the showerhead itself is circular. Secondly, a symmetrical pattern of the spray holes does not necessarily mean spray symmetry due to imperfections of individual spray holes in terms of size and orientation. Thirdly, the presence of showerhead installation angle changes the spray pattern due to gravity and air friction. Even if the spray is perfectly symmetrical, it is very difficult to ensure the spray centre coincides with that of the collector unit if possible. Finally, as can be seen in Table 6 in the Appendix, showerheads come in different forms and shapes in addition to the conventional circular base. As such, a collector system with circular zones cannot serve to map the actual spray distribution.

**Fig. 2:**
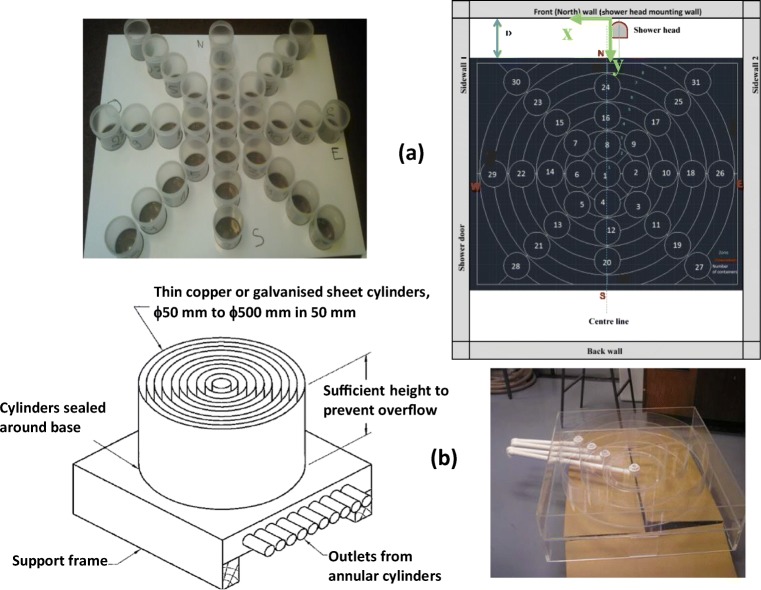
Spray collector units (**a**) current study, with plan view and (**b**) Alkhaddar *et al.* (2007)

Ideally, a refinement to the circular zone design should involve each circular zone being sub-divided into smaller zones. This however would significantly increase the manufacturing and operational difficulties. The adopted spray collector design overcomes the asymmetry issue by using a series of plastic containers (cups) arranged in the configuration shown in Fig. 2a.

The proposed spray collector design makes it possible to estimate the central spray position using the measured spray volumes of individual cups. With the positions of the cups defined in terms of *x*-*y* coordinate (refer to Fig. 2a), the centre of the spray was calculated as the centre of gravity of the measured volumes:

$$ {x}_c=\frac{\sum {V}_i{x}_i}{\sum {V}_i},\kern1.25em {y}_c=\frac{\sum {V}_i{y}_i}{\sum {V}_i} $$

where (*x*_*i*_, *y*_*i*_, *V*_*i*_) represented the position (*x*_*i*_, *y*_*i*_) and volume (*V*_*i*_) of the ith cup. Not all cups were included in the calculation. A measurement is included if *V*_*i*_ ≥ 0.3*V*_max_, where *V*_max_ is the maximum volume per cup collected in a showerhead test. This decision was because all the tested showerheads had a spray collector coverage of at least 70% of the total spray area. Excluding the low-volume cups which has the effect of alleviating potential bias and inaccuracy caused by low spray water area that extends outside the bounds of the collector unit.

A precision template was laser-cut from an 8-mm Perspex sheet, ensuring repeatability and consistency of the container positions between tests of the different showerheads. The position of the collector unit in the shower cubicle is shown in Fig. 2a. Initial trials showed that depending on the showerhead being tested, the centre of the shower spray was generally not the same as the centre of the container array. For consistency and improved spray area coverage, each showerhead was tested three times with the template placed at 0.55 m, 0.60 m, and 0.66 m from the north wall, respectively. In addition to the increased spray area being measured, the gapping area between the container arrays were also covered. The spray collector unit serves to obtain the flow distribution and temperature variations across the spray. For each test, water was dispensed for 60 sec. During this time, the collected water did not overflow in any of the cups. This made it possible to calculate the relative volume of the spray at these positions with respect to the total amount of water released from the showerhead. The total flow rate was measured through an in-line propeller flowmeter and the data was recorded by the data logging system, whereas the relative volume of water in the cups was derived using a digital scale.

### Showerhead temperature range

Thermal imaging is now widely used in the construction industry and for the purposes of this study, temperature differentials can be used to define temperature ranges and heat transfer from water being dispensed from the showerhead. Thermal imaging cameras work by recording infrared radiation (IR) which directly relates to the objects’ temperature.

In using thermal imaging cameras in this way, reflections and emissivity of the object may be considered. In this case, the thermal camera with emissivity factor of 92 was used. The emissivity was defined by the following relationship:

$$ \varepsilon ={\phi}_R/{\phi}_B $$

where *ε* is the emissivity factor, *ϕ*_*R*_ the emissivity of the object or the fluid, and *ϕ*_*B*_ the emissivity of the blackbody at the same temperature.

The Thermo View Ti30 thermal imaging camera by Raytec was used. It had a supporting software package that converts recorded thermal images into temperature data. The water temperature at each cup was obtained at the end of each test. Four thermal images were taken, each covering 1/4 of the container array and together provide the temperature information across the whole collector area.

### Showerhead pressure array

**Fig. 3:**
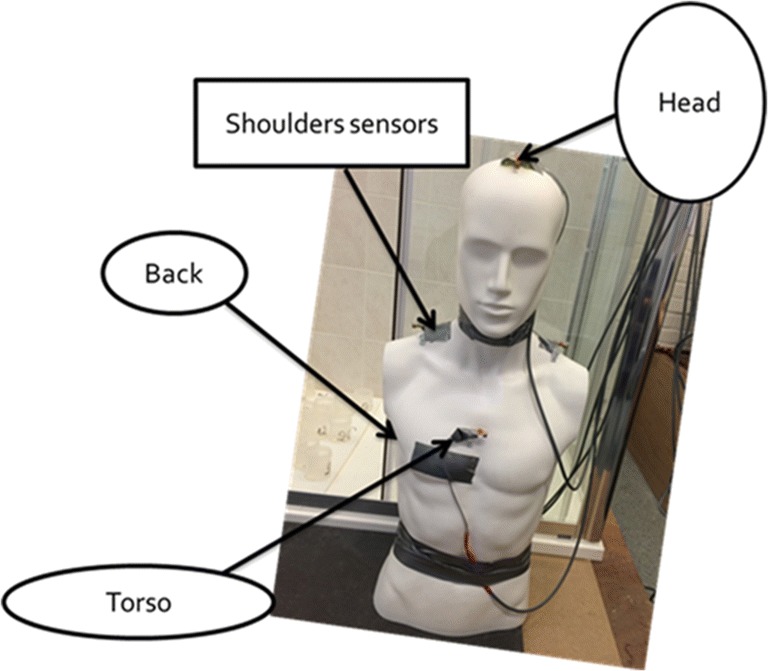
Setup of pressure sensors

The body-contact pressure sensor array consisting of an array of five pressure sensors was mounted on a mannequin as shown in Fig. 3. Only the upper body of the mannequin was used, which sits on a custom-made tripod support such that it could be rotated easily. The overall height of the mannequin was 1.8 m. The sensors were positioned on the forehead, the shoulders, the chest, and the back of the body. These are the body positions most sensitive to the spray pressure. The pressure sensor array gauges the “feel” of the pressure on different parts of our body when facing the spray. The body pressure measurements were recorded separately to the flow distribution tests.

### User feedback

A 2-stage participant sampling process was then utilised. The first stage consisted of publicising the challenge to students and colleagues working in the various campuses of a university and to residents in the immediate vicinity. This was done via university mailing lists and through a gatekeeper for the campus neighbouring areas. Interested participants were asked to complete a brief online survey, and the findings were published in Adeyeye and She (2015). About 200 responses were received. From this, 12 participants were sampled for the second stage based on the age, gender, anthropometrics, property tenure, bathroom, and water fittings set-up, and logistical issues such as the ability to commit to a 12-week study, distance, and property access.

A pre-commencement workshop was held to formally introduce the participants to the aim and objectives of the project, to issue the study kit, which included shower timers, and to discuss information and feedback requirements. The workshop was also used to establish a weekly diary for delivering the showerheads and collecting the feedback sheets. The study then commenced the following week at the beginning of April and concluded 3 months later in June. This duration and season should be considered in the interpretation of the results. The findings were analysed over a 2-week period. A post-study workshop was then organised with the same set of participants to feedback the findings and clarify gaps/questions in the data. Nine of the 12 participants attended. At the end of the workshop, participants were given the option to choose and keep one of their top three preferred showerheads as the incentive for participating in the study.

## Results: uses and users

Prior to the post-use user feedback, some general demographic information (age, gender, etc.) and the broad factors, that influence the choice and purchase of the showerheads by the participants, were collected. The key decision parameters are summarised in Table 2. Majority of the participants prioritised the performance—the spray pattern of the showerhead—as an important decision criterion. They perceived this to be due to the number, rather than the type of spray sprouts. Interestingly, majority of participants did not consider the eco-credentials or cost of the showerhead, or the cost of water as important decision factors. Table 2 should be considered within the context of the limited sample size of this study.

**Table 2:** Parameters influencing the participants’ choice and purchase of showerheads

Decision factors		Cost of showerhead	Cost of water	Showerhead design	Eco-credentials/ratings	Showerhead material	Showerhead size	Showerhead pressure	Spray pattern	Number of sprouts	Type of sprouts	Feel of showerhead	Known performance, e.g., word of mouth	Previous experience
Freq (%)	Yes	16.7	16.7	33.3	0	33.3	16.7	33.3	66.7	50	0	0	0	16.7
	No	83.3	83.3	66.7	100	66.7	83.3	66.7	33.3	50	100	100	100	83.3

**Fig. 4:**
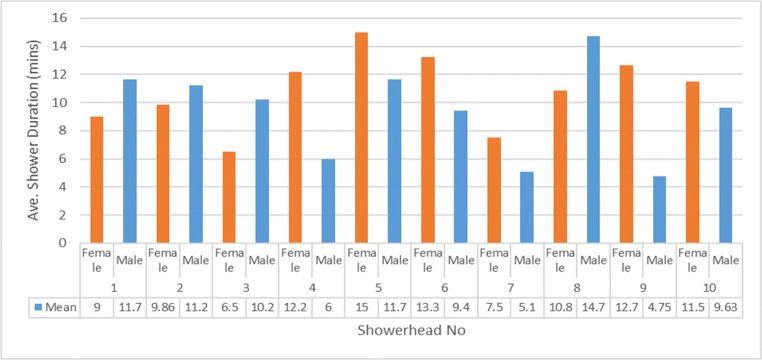
Mean shower duration per gender

Participants were given shower timers to record their shower durations. Figure 4 and Table 3 compare the perceived and actual shower duration based on the gender of the participants. It was found that the female participants in general perceived that they spent more time in the shower, compared with men. The shower duration varied depending on each showerhead. There were differences between perceived and actual shower duration for different showerheads as follows: (i) compared with the male participants, the female participants spent less time in four of the 10 showerheads, although women in general perceived themselves to spend more time in the shower . (ii) Men spent more than 10 min, on average, in 5 of the 10 showerheads, although perceiving that they never had long showers.

**Table 3:** Duration of shower per time of day and gender

Time of day	Duration	Gender (% response)	
		Female	Male
Morning shower (before noon)	Long (more than 10 min)	100	0
	Moderate (5–10 min)	0	75
	Quick (less than 10 min)	0	25
Afternoon shower (noon to 6 pm)	Long (more than 10 min)	100	0
	Moderate (5–10 min)	0	100
Evening shower (after 6 pm to midnight)	Long (more than 10 min)	50	0
	Moderate (5–10 min)	50	100

The participants were also asked to categorise the purpose of their shower as follows: daily hygiene, to refresh (e.g., after a sporting activity), or to relax (e.g., after a workday). The purpose of the showers again varied depending on the time of day or day of the week (Table 4). The data further highlights that for this group, there were more variety in the purpose of the shower during the weekends.

**Table 4:** Purpose of shower per time of day/day of the week

Purpose of shower	Day of the week (% response)							Time of day (% response)		
	Monday	Tuesday	Wednesday	Thursday	Friday	Saturday	Sunday	Morning	Afternoon	Evening
Hygiene—daily routine	100	100	100	100	100	66.7	80	100	-	
To refresh—e.g., after physical activity, to wash hair, or before evening socials	-	-	-	-	-	16.7	20	-	66.7	50
To relax—e.g., after a workday	-	-	-	-	-	16.7		-	33.3	50

Two stages of feedback were obtained: one on receipt of the showerhead at the beginning of the trial week and latest after first use (referred to as pre-feedback in Table 5), and second at the end of the trial week after the final showering event before the swap of showerheads (referred to as post-feedback in Table 5). Feedback were provided on a scale of 1 to 5, and the notional mean for acceptable, positive feedback was defined as 3.5. The average user feedback is shown in Table 5 (Upright—above notional mean, Italics—below notional mean). It should be noted that showerheads 7 and 10 are identical in all aspects except for the colour (see images in Table 6 in the Appendix). This was done as an additional indicator of consistency in the user feedback of each product. It was observed that, although the answers are not always similar, the answers for showerhead 10 are in general closer to those of showerhead 7 than to any other showerhead.

**Table 5:** All pre- and post-use feedback from study participants. Mean figures shown

Metrics	Coded feedback (pre and post user trial)	S-1	S-2	S-3	S-4	S-5	S-6	S-7	S-8	S-9	S-10
Consistency of flow	Pre use_Consistentflow	4	4.27	4	4.2	4.38	*3.33*	4.38	4.17	4.1	4.5
	Post use_Consistentflow	4	4.42	4	4.2	4.36	*3.44*	4.13	4.08	4	4.42
Consistency of pressure	Pre use_Consistentpressure	4	4.09	4	4.3	4.42	*3.22*	4.38	4.25	4.1	4.5
	Post use_Consistentpressure	4	4.5	3.88	4.1	4.36	*3.44*	4.25	4.08	4	4.42
Pressure (force impact) on body	Pre use_Pleasantpressure	*2.89*	3.73	*3.22*	3.9	3.62	*3.11*	4.13	3.58	3.6	4.08
	Post use_Pleasantpressure	*3*	3.83	*3.13*	3.9	3.82	*2.89*	3.88	3.58	3.5	4.08
Feel of the spray	Pre use_Spraypressure	*3*	3.82	*3.33*	4	3.69	*3.22*	3.75	3.67	3.9	4.17
	Post use_Spraypressure	*2.78*	3.75	*3*	4.1	3.36	*3.11*	3.75	3.67	3.7	4.25
Coverage of the spray	Pre use_Spraycoverage	*2.67*	*3.36*	3.56	4.2	3.62	*3.33*	3.63	*3.33*	*3.2*	4
	Post use_Spraycoverage	*2.56*	*3.33*	3.5	4.2	3.64	*3.11*	4.13	*3.33*	*3.4*	3.91
Acoustics (pleasant and soothing sound)	Pre use_Sound	*3.44*	4	3.89	3.9	3.38	3.56	3.88	3.83	4	4.33
	Post use_Sound	*3.22*	3.92	3.75	3.6	3.73	*3.22*	3.75	3.83	3.8	4.17
Consistency of temperature	Pre use_Temperature	4	4.55	3.89	4	4.08	3.67	4.13	3.83	4.2	4.25
	Post use_Temperature	3.89	4.33	3.75	4.2	4.09	*3.44*	4	3.83	4.1	4.33
Changed shower habit to suit showerhead	Pre use_Changehabit	*2.78*	*2.36*	*2*	*2*	*2.85*	*3.33*	*2.88*	*2.75*	*2.1*	*2.67*
	Post use_Changehabit	*3.11*	*2.67*	*2.13*	*2*	*3.27*	3.67	*3*	*3*	*2.5*	*2.5*
Had to change body positioning in the shower	Pre use_Changeposition	*2.89*	*3.18*	*2.56*	*2.1*	*3.38*	3	*3*	*3*	*2.4*	*2.83*
	Post use_Changeposition	*3*	*3.17*	*2.75*	*2*	*3.09*	*3.11*	*3.29*	*3.25*	*2.2*	*3*
Ease of Use	Pre use_Ease	4.22	4.25	4	4.3	4.23	*3.44*	4.25	4	4.2	4.5
	Post use_Ease	3.67	4.42	4.38	4.4	4.55	*3.44*	4.63	3.83	4.4	4.42
Enjoyment during use	Pre use_Enjoyableuse	*2.89*	3.64	*3.44*	3.8	3.85	*3.33*	4	3.67	3.8	4
	Post use_Enjoyableuse	*2.78*	3.67	*3.25*	4.1	*3*	*3*	4.13	3.75	3.7	3.92
Look (design)	Pre use_Look	3.56	*3.08*	*3.33*	4	3.69	*3.44*	4.38	4	3.7	4.33
	Post use_Look	*3.33*	3.5	*3.38*	4.1	3.64	3.78	4.38	4	3.6	4.33
Effort to clean e.g. hair	Pre use_Loweffortclean	*2.67*	*2.27*	*2.33*	*2.7*	*2.62*	*2.67*	*2.75*	*3*	*2.7*	*2.67*
	Post use_Loweffortclean	*2.11*	*1.83*	*2.38*	*2.9*	*2.73*	*2.78*	*2.75*	*3.25*	*2.4*	*2.83*
Showered more than usual	Pre use_Morefrequse	*2.67*	*2.36*	*2.89*	*2.4*	*2.15*	*2.11*	*3.38*	*2.58*	*2.8*	*2.42*
	Post use_Morefrequse	*2.67*	*2.25*	*2.13*	*2.3*	*2.55*	*2*	*3.13*	*2.75*	*2.6*	*1.92*
Physical feel (handling)	Pre use_Physicalfeel	*2.78*	*3.09*	*3*	3.6	*3.08*	*2.89*	3.75	3.5	*3.3*	3.92
	Post use_Physicalfeel	*2.89*	*3.17*	*3*	*3.3*	*3*	*2.56*	4.25	3.58	*3*	3.83
Quickness (ease) to install, stay as installed	Pre use_Quicktoinstall	4.67	4.55	4.11	4.6	4.38	4.33	4.75	4.67	4.5	4.75
	Post use_Quicktoinstall	4.56	4.67	4.38	4.7	4.55	4.44	4.75	4.83	4.6	4.58
Performs as expected	Pre use_Worksasexpects	3.56	3.55	3.89	4	3.69	*3.33*	3.75	3.58	3.9	4
	Post use_Worksasexpects	*3*	*3.33*	3.88	3.8	3.36	*3.11*	3.63	3.75	3.7	3.92
Continuity of use	Pre use_Continueuse	*2.67*	*3.18*	*3*	3.9	*3.23*	*2.89*	3.63	*3.25*	*2.8*	3.5
	Post use_Continueuse	*2.22*	*2.67*	*2.75*	3.6	*2.64*	*2.56*	3.88	*3.25*	*2.8*	3.75
Willingness to buy	Pre use_Happytobuy	*2.44*	*3*	*3*	4	*2.92*	*2.56*	3.63	*3*	*2.4*	3.5
	Post use_Happytobuy	*2.33*	*2.67*	*2.75*	3.8	*2.36*	*2.44*	3.75	*3.08*	*2.8*	3.5
Meets all expectations	Pre use_Meetsexpectats	*2.33*	*2.91*	*3*	3.8	*3.08*	*2.78*	3.75	*3.08*	*3.2*	3.58
	Post use_Meetsexpectats	*2.33*	*3.25*	*2.75*	3.7	*2.64*	*2.78*	3.75	*3.17*	*3.2*	3.67
Preference compared to pre-study showerhead	Pre use_Preferencecompare	*2.33*	*2.18*	*2.44*	*3.2*	*3*	*3.11*	*3.13*	*3.25*	*2.6*	*2.83*
	Post use_Preferencecompare	*2.22*	*2.42*	*2.5*	*3.3*	*2.82*	*2.89*	*3.29*	3.5	*2.4*	*3.08*
Likelihood to keep	Pre use_Willkeep	*2.33*	*2.73*	*2.44*	3.8	*3*	*2.44*	*3.38*	*2.83*	*2.3*	*3.42*
	Post use_Willkeep	*1.78*	*2.25*	*2.63*	3.5	*2.36*	*2*	3.57	*3.17*	*2.5*	3.5
Personal preference for this showerhead compared to the others	Pre use_Worksforme	*2.78*	*2.73*	*3.22*	3.9	*3.23*	*2.78*	3.88	*3.08*	*2.7*	*3.42*
	Post use_Worksforme	*2.33*	*3.08*	*3*	3.9	*2.64*	*2.56*	3.63	*3.17*	*3.2*	3.58

For each showerhead, the participants gave a rating against each statement based on a choice of five options: strongly disagree, disagree, neutral, agree, and strongly agree. The statements and rating options were conveniently presented in the form of a table for ease of use and were explained to participants at a commencement workshop to ensure that the feedback statements were interpreted correctly when completing the feedback sheets. During the 10-week shower challenge, participants were asked to complete a feedback sheet, which documented their showering habits (when they showered and why), as well as their feedback against the twenty three statements relating to the showerhead design, appearance, perception of spray, and experience of use. These metrics were used to gauge both the preference and satisfaction with each trialled showerhead. It was found that users were able to discern differences in the showerhead performance in terms of the consistency of flow and temperature as well as the spray coverage. Further, user judgement and experiential perceptions of the showerheads differed throughout the trial. Therefore, and depending on the showerhead, the mean value for the showerhead increased or decreased throughout the trial. This suggests that there could be significant differences in findings from water efficiency studies that are based on experiential data compared with findings from other studies where the users did not have the opportunity to trial the showerheads.

All the showerheads except showerhead 6 (see images in Table 6 in the Appendix) performed above the notional mean on consistency of water flow, pressure, water temperature and on ease of use. Showerhead 6 is a round showerhead with recessed twin sprout types laid out as 3 × 3 double-sprout clusters. This shower delivered the least regulated flow rate of 5.1 l/min @ 2-bar pressure of all the showerheads and had the least number of sprouts. It was also the least favoured in the feedback about the shower pressure and physical feel at the end of the trial week (Table 5, see Tables 7 and 8 in the Appendix for results per gender). Showerhead 1 also performed poorly despite the higher flow rate. This showerhead was the other round showerhead of the sample with recessed twin sprout types laid out as 3 × 3 double-sprout clusters and had the lowest feedback values for feel of the spray, spray coverage, and enjoyment during use.

The participants gave close values for the pressure on body and feel of the spray for each showerhead, suggesting that it was difficult to dissociate one feeling from the other. In fact, only showerhead 5 performed above the notional mean regarding pressure on the body and below the notional mean in terms of feel of the spray. Concerning spray coverage, showerheads 1, 2, 6, 8, and 9 all performed below the notional mean. Showerheads 1 and 6 are round and 2 is oblong but does rotate whilst 8 and 9 are rectangular. The showerheads 1, 2, 6, and 9 have a similar sprout design—recessed twins. Whilst showerhead 8 has triple central, recessed twin arranged from the centre in triple clusters and random rows (see images in Table 6 in the Appendix). They deliver regulated flow rates of 8.7, 8.7, 5.1, 7.3, and 8.3 l/min @ 2-bar pressure respectively. All 5 showerheads (numbers 1, 2, 6, 8, and 9) work by delivering colliding twin jets of water that turn into thousands of tiny droplets.

**Fig. 5:**
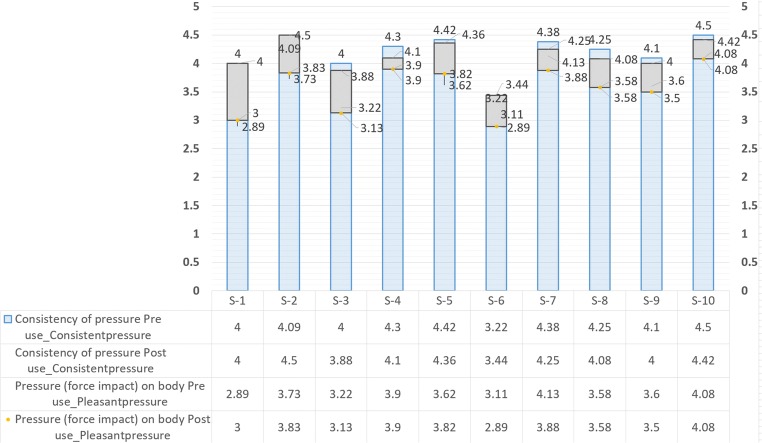
Mean feedback on showerhead pressures

The response for the feel of water pressure on the body whilst showering was also interesting (Fig. 5). Free text feedback supported the returned means for showerheads 1 and 6 especially at the end of the trial week. The sprout type, layout, and mode of operation of these two showers meant that water was delivered as a spray. The participants, especially women, stated that this made cleaning and hair washing difficult. Both showerheads also only had one function, although this is not significant as not all showerheads with multiple function performed significantly better.

All the showerheads were rated well above the notional mean for consistency of temperature at the beginning of the trial. The feedback for showerheads 4 and 10 increased at the end of the week, the others remained close, except for showerheads 1 and 6, which experienced higher negative change. Showerhead 6’s feedback also dropped below the notional mean at the end of the user trial. Showerheads 4, 5, 7/10, and 9 were the better performing showerheads for this variable. Showerheads 4 and 5 have similar design but 5 has more sprouts and is distinct in its mode of operation; showerhead 5 has one function unlike the similar showerhead 4, which has three functions. Like showerhead 4, it has a protruding sprout arranged in radial rows around a central core. Their mode of operation is also significantly different from the others as they are the only two showerheads that mix water with air; hence, some participants complain that they are noisy.

Showerheads 7 and 10 ranked highest for enjoyability at the beginning of the trial—they were the largest showerheads in the sample and had just two functions, which were obvious and easy to use. No price information was provided to the participants as price factors were outside the scope of the study. Therefore, the participants were unaware that showerhead 2 was the most expensive multifunctional showerhead in the sample. Although this showerhead performed above the notional mean for the technical metrics, e.g., temperature and pressure, it did not for preferential criteria like effectiveness for cleaning, handling, and willingness to buy. It was also found that most of the participants did not identify that it had multiple functions until they were told at the end of the challenge, as these functions were not obvious or intuitive. At the end of the trial, this and showerhead 4 were considered the most enjoyable. In agreement, the participants stated that showerheads 4, 7, and 10 met their expectations and were the only showerheads that performed above the notional mean regarding the willingness to buy and keep using, showing a consistent preference for the showerheads. Interestingly, as in Wong et al. 2016, the participants would prefer to keep their original showerheads than change to one of the trialled (only showerhead 8 performed above the notional mean at the end of the trial period for this variable). This might be partially due to preferences and habits or perceived relative performance. Nevertheless, it was observed that this preference might change with time or longer period of use. In fact, except for showerheads 4 and 10, the mean value for the showerheads increased throughout the trial regarding the need to change shower habits to suit the showerhead. Also, considering the feedback for the flow, spray distribution, and temperature of the showerheads combined, it was deduced that showerheads 4, 7, and 10 received the most positive responses from the participants. However, for flow only, showerheads 2 and 5 were considered better than 4; for temperature, showerhead 2 was considered better; and showerheads 5 and 9 had a similar feedback to showerhead 4.

These findings in addition to those presented in Adeyeye et al. (2017) affirm the complexity of user perceptions and experiential use of showerheads. It also shows that multiple design and performance considerations determine the degrees of suitability, adoption, satisfaction, and water use efficiency of products such as showerheads.

## Results: laboratory results

**Fig. 6:**
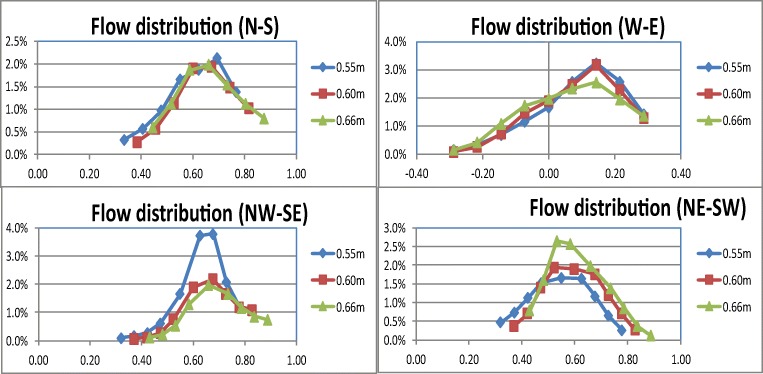
Variation of spray volume as a function of floor position for showerhead S-1

**Fig. 7:**
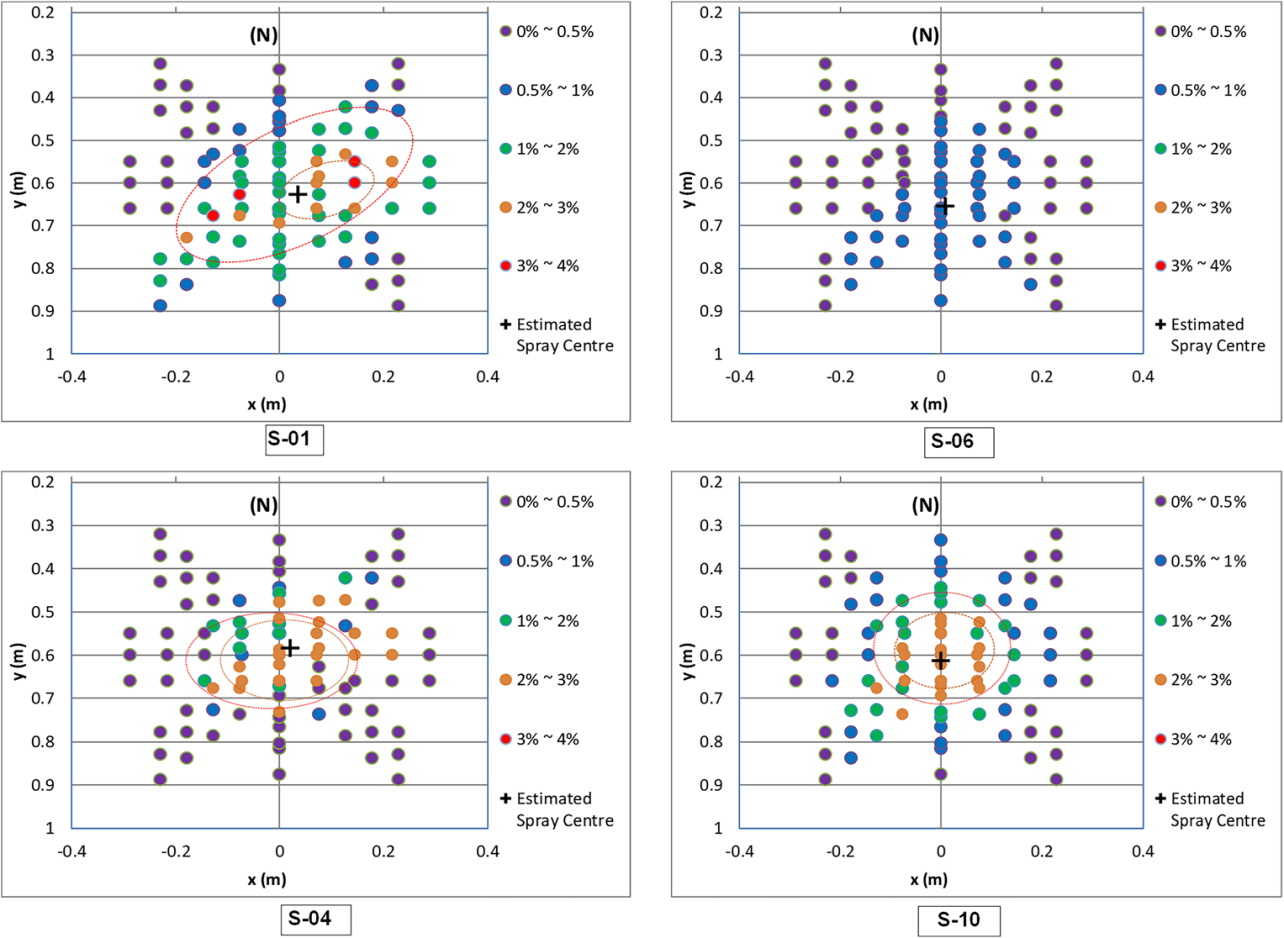
Spray distribution for showerheads 1 and 6 of low overall ratings, 4 and 10 of high overall ratings

As postulated, no clear evidence that showerhead spray is symmetrical with respect to a central point was found (Figures 6 and 7). The spray volume measurements indicated that the per-cup volume is up to 4% of the total spray volume. For mapping the spray distribution, the measured per-cup volumes were grouped into five ranges of 0.001–0.5%, 0.5–1%, 1–2%, 2–3%, and 3-4% (Fig. 6). These are colour-coded and shown along with the cup positions, thus providing a visual indication of the varying spray intensity over the spray area.

The spray water distribution in different directions are shown in Figure 7 (North-South, East-West, etc., refer to Fig. 2a). The spray water measurement was repeated 3 times for each showerhead with the spray collector unit respectively placed at 0.55 m, 0.60 m, and 0.66 m from the north wall. Take special note of the row of cups in the N–S direction formed of cups 24-16-8-1-4-12-20. An overlap was found when moving the collector unit between tests. The cups on the overlapped positions should therefore give similar measurements despite the shift of collector position. Good consistency and repeatability were observed between the tests in the different collector positions when the results were examined in the N–S direction.

**Fig. 8:**
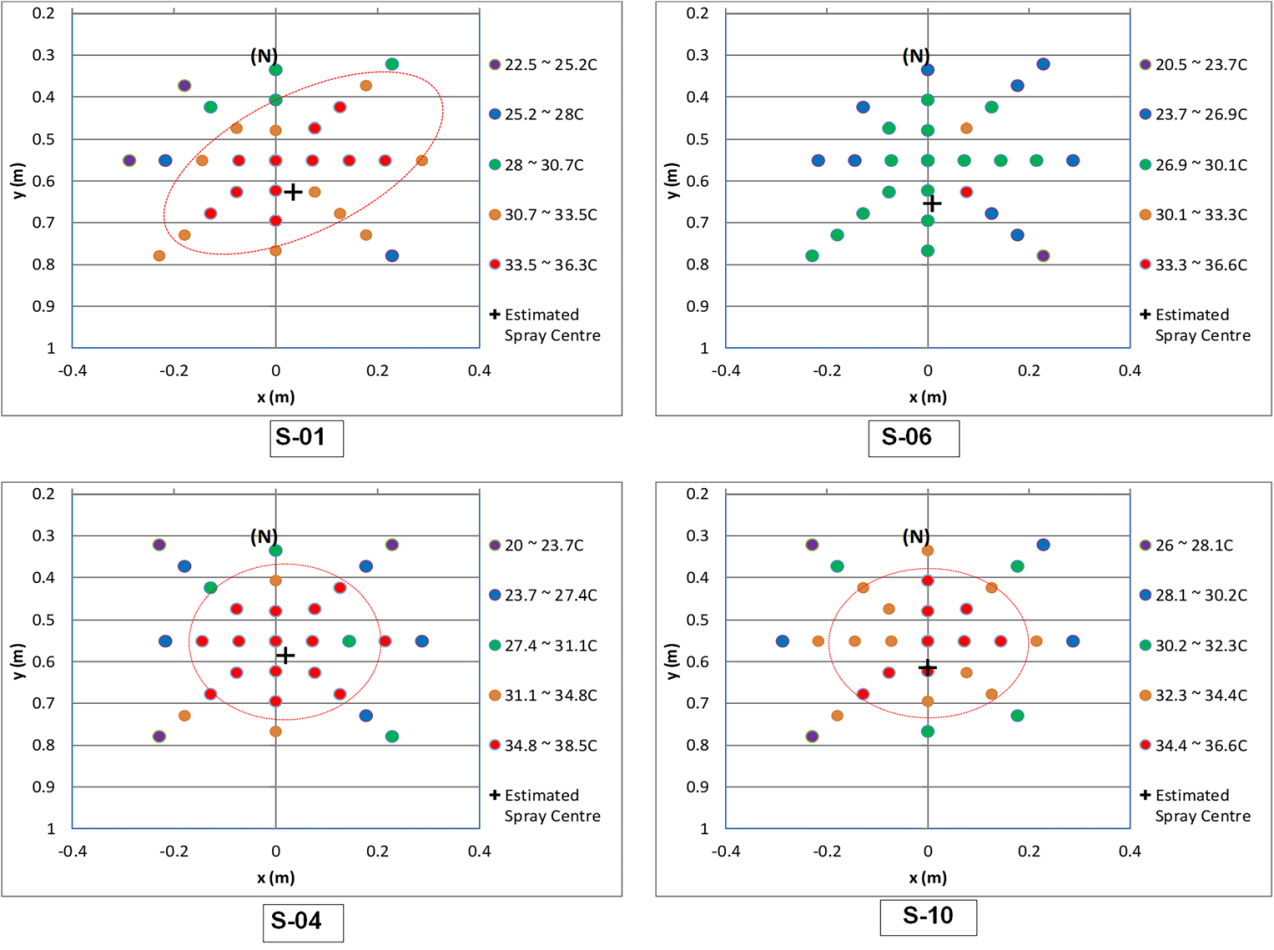
Spray temperature distribution for showerheads 1 and 6 of low overall ratings, 4 and 10 of high overall ratings

Findings from the user feedback were that showerheads 4 and 10 attracted some of the highest overall ratings while 1 and 6 received the lowest. Flow measurements for these showerheads are presented in Fig. 8 which mapped the volumetric distribution over the spray area. The mapping provides an insight into three aspects of the spray, i.e., spray form or pattern, magnitude and variation of spray intensity, and effective spray area or coverage. It was observed that the spray form is generally non-symmetrical with respect to the centre of the spray irrespective of the showerhead design. This agrees with the previous discussions in the “Methods” section with respect to the spray collector design. Note that showerhead 4 is a circular shape with a radially symmetrical distribution of spray holes but the actual spray water distribution is still asymmetric just like other showerheads of different designs.

To view the spray pattern, dashed curves approximately show the central areas where the collected spray water exceeds 1% (in red) and 2% (in orange). Showerhead 10’s pattern had the nearest to radial asymmetry, followed by showerhead 4. The percentage volume of water collected in each cup was viewed as the intensity of the flow at the cup position. Showerheads 4 and 10 both have a clearly defined central area where the spray intensity is above 2%, extending outward to the 1% region. It is visible that the high intensity (above 2%) occupies the central spray area with a small 1% intensity band. In contrast, showerhead 1 has a very small area above 2% with a large extended region of 1% intensity. In addition, high-intensity points are dotted within lower intensity areas, which indicates a high degree of uneven distribution of the spray water.

The spray intensity has significant implications on the spray pressure felt on the body. A higher intensity of spray means a higher flow rate and velocity over the cup area. A higher flow velocity in turn means a greater impact force felt on the body. This results from the basic momentum principle, which stipulates that the impact force of the water jets on the skin is directly proportional to the flow rate and velocity. The above statement assumes a direct elastic impact, which is not strictly true. Nevertheless, it is true that the higher the intensity, the greater the impact force. Showerhead 6 has a spray intensity at below 1%, which is significantly lower than all other showerheads. Showerhead 1 contains a high-intensity area of 2% but the area is significantly smaller than showerheads 4 and 10 (Fig. 8). Therefore, it can be deduced that an adequate area of good spray intensity is needed to give satisfactory user experience.

**Fig. 9:**
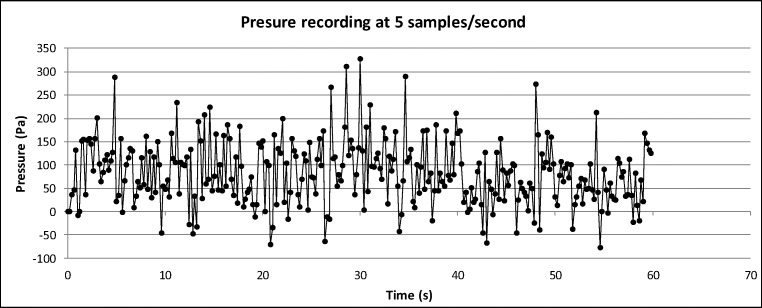
Typical time series of pressure measurement

Like spray volume distribution, the temperature of the spray was mapped, and this is shown in Fig. 9 for showerheads 1, 4, 6, and 10. As an example, the temperature analysis contains one data set for each showerhead. Still, the trend and general behaviour could still be observed, and it does mean less detail compared with the spray volume mapping exercise. Following initial analysis, the temperature mapping was carried out by setting five zones between the maximum and minimum readings. It is interesting to note that the temperature variations follow a similar pattern as the spray volume distribution. The red-dashed enclosure indicates the area of 1% spray intensity. There is a drop of about 2 °C between adjacent zones, indicating significant heat transfer from the spray to the surrounding air. It was also apparent that showerhead 6 had only two cups in the two highest temperature zones, indicating that heat transfer of this showerhead is higher and faster than the others. This means that this showerhead, while being the most water efficient, offers poor spray intensity and heat retention. Temperature variation for showerhead 1 showed no obvious difference to 4 and 10 apart from the stated asymmetry. Thus, it appears that the thermal characteristics of the spray may have an influence on the user’s showering experience, but the spray volume distribution has a dominant effect.

**Fig. 10:**
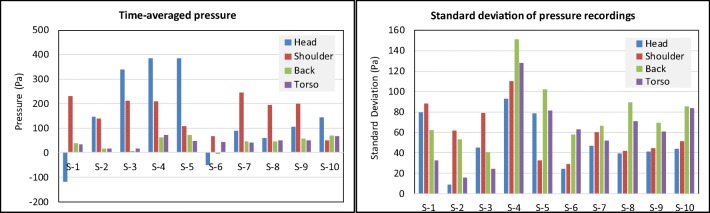
Comparison of measured pressure at different body positions

As shown in Fig. 3, force transducers were mounted at different positions of the mannequin to provide a direct measurement of impact pressure on the body when taking the shower. A typical time series of the findings are shown in Fig. 9. The impact force on the measuring disc was highly unstable due to the unsteady nature of the shower spray, thus leading to large fluctuations of the recorded data. As a preliminary result, Fig. 10 shows the mean pressure along with the calculated standard deviations. There were significant fluctuations for all showerheads at all body positions. It is noteworthy that showerheads 1 and 6 showed negative pressure values at the head position. This does not really mean negative pressure, but it is an indication of inadequate spray jets hitting the measuring disc in this location. Thus, creating an extremely uneven pressure distribution. The reason is that the impact pressure is measured by means of a load cell rigidly mounted to the centre of the measuring disc. The use of high-sensitivity load cells allowed the detection of very low pressure of the spray but there was potential loss of accuracy if a significant bending moment is present. In the case of showerheads 1 and 6, there are only a small number of spray holes; the load cell at the head position is in proximity of the showerhead such that the disc area only catches one or two spray jets. This led to adequate bending moment that causes the observed negative pressure readings. Further investigations are needed to ascertain the quantitative effect of uneven pressure distribution in order to determine an optimum disc size.

Broadly speaking, the magnitude of impact pressure reduces with lower body positions. This is primarily caused by an increase in the spray area. Overall, showerhead 4 showed the highest pressure readings across all the body positions followed by 5, 7, 8, 9, and 10. Showerhead 3 had high readings at head and shoulder levels but low at back and torso. Showerheads 6 and 1 had the lowest readings and/or uneven distribution of impact pressure force. This suggests that the magnitude of impact pressure and evenness of spray distribution influence the user showering experience and thus their preference of showerheads.

The findings can be summarised as follows. In descending order, the 5 showerheads least prone to be bought: showerheads 1, 5, 6, 2, 3; used: showerheads 1, 6, 5, 2, 3; and kept: showerheads 1, 6, 2, 5, 9. Considering these 3 metrics, showerheads 1 and 6 are the least preferred by the participants. They are also the showerheads with lower grades in terms of pressure on body and feel of the spray, at both the beginning and the end of the trial week, suggesting that these characteristics are the most important for the participants. Consistently, these are the showerheads where the laboratory experiments showed lower measured pressure in the head and non-uniformity of the temperature distribution or high loss of energy across the sprouts. The participants had more willingness to buy these 5 showerheads (in descending order): showerheads 4, 7, 10, 8, 9; use: showerheads 7, 10, 4, 8, 9; and keep: showerheads 7, 4–10, 8, 3. Considering these 3 metrics, showerheads 4, 7, and 10 were the most preferred by the participants, with 7 and 10 having the same characteristics but the colour. These showerheads received the highest mean values in terms of pressure on body and coverage of the spray, at both the beginning and the end of the trial week, suggesting that these characteristics are the most important for the participants. These showerheads were also in preferred for their better spray feeling, along with showerheads 2 and 9.

Overall, showerheads 4, 7, and 10 were considered the showerheads that meet all expectations, even if showerhead 4 forced the participants to change shower habits and to change body positioning in the shower, showing that the participants value pressure, spray coverage, and spray feeling, willing to change their showering habits if necessary, in order to improve those 3 metrics. Comparing with lab experiments, showerheads 4 and 10 have reasonable radial asymmetry of the spray. Regarding measured pressure, showerheads 1 and 6 showed the lowest pressure in the head and showerheads 2 and 3 the lowest in the back and torso, comparing with showerheads 4, 5, 7, 8, 9, and 10, the measured pressure cannot explain by itself the preference of the participants for showerheads 4, 7, and 10. From the user feedback, the 10 showerheads were clustered into two distinct groups; preferred and not preferred. Showerheads 1 and 6 were distinctly not preferred compared with the others. Showerheads 1 and 6 have similar designs, with less sprouts compared to the others, and deliver water as fine sprays. These findings were checked against the perception of spray pressure on the body during the shower and there was correlation in the findings.

## Discussion

Water use reduction or enabling more efficient water use is necessary to maintain long-term water supply and security (Lede and Meleady 2019). Thus, the use of water-efficient appliances has been widely regarded as the best way to unconsciously save water (Batchelor et al. 2014). Therefore, previous studies have explored the sociological and practical reasons for the increase in water use in the shower and suggested technological, behaviour change, and information strategies for increasing water efficiency. The number of field experiments on how people’s behaviour can be changed with respect to their daily water consumption is growing. But to date, most studies in this field have focussed either on explanatory socio-economic factors (e.g. water pricing, income, or family composition) or behavioural intentions and personal characteristics related to behavioural change (Koop et al. 2019). Further, studies on the merit of technological interventions have yielded diverse results. For example, studies on water-efficient showerheads combined with feedback on use such as Wong et al. (2019); Kenney et al. (2008) found 10% water savings, whilst another , Stewart et al. (2012) found reduced shower’s duration by almost 30% but that shower water use returned to pre-installation levels after 4 months. Therefore, despite numerous empirical studies, key knowledge gaps remain about the influences on household water consumption (Manouseli et al. 2019). These gaps include the comprehensive understanding of how individual characteristics, as well as energy and water-efficiency labelling schemes, affect conservation behaviours and the factors that affect household decisions about the adoption of energy- and water-efficient appliances (Dieu-Hang et al. 2017). At present, water efficiency labelling and product standards place emphasis on showerheads to discharge water at certain flow rates—typically 9 l per min. This oversimplification contradicts research findings that multiple factors affect user satisfaction with environmental products, as consequently their response and use of such products.

No study has fully explored the performance parameters that define the degree to which products are accepted or rejected. It was therefore considered useful to deconstruct the showering process to determine how best to design and target technological solutions to save water while meeting the performance expectations of user. It builds on the work reported in Adeyeye et al. (2017), which studied the sustainability mismatch between design/performance metrics of the sampled showerheads on one hand and user feedback of experience on the other. Based on this, this paper focused on flow and temperature parameters as two main parameters that can be used to understand user preference of one showerhead compared with the other, but also to understand how this preference is defined by their experience of the product.

This study found good merit in including the spray intensity (pressure) and distribution and degree of heat loss, in addition to the discharge rate, as part of showerhead performance and efficiency considerations. Further research is needed to determine the optimum spray intensity and distribution and the findings should be considered within the limitations of the sample size. The findings support that of Okamoto et al. (2015a and b) which studied quantitative showerhead factors as influences on satisfaction and found that the spray force, spray force-per-hole, spray pattern, water volume ratio in spray patterns within φ100 and φ150, temperature drop, and spray angle all influenced satisfaction. They proposed that the physical properties of spray force-per-hole and temperature drop, and designs that set an appropriate value for water distribution and spray angle for spray patterns within φ100 and φ150, improves satisfaction independent of usage water flow (Okamoto et al. 2015a). They concluded that satisfaction and usage water flow have a spurious correlation relationship, and this was also confirmed by this study. But the findings contradict Meireles et al. (2018) study of taps, which found that female participants were more conservative in estimating their use duration and therefore water use compared with the male participants. This study did not find such gender correlations; instead, the shower durations varied more in line with the use, and effectiveness of the showerheads to meet the participant’s requirements and levels of experiential satisfaction, relative to the purpose of the shower.

As this was a socio-technical study, it was important to compare the user feedback against findings from the laboratory tests. Again, the underlying link between the spray characteristics and user perception of shower quality appeared to be confirmed. The less rated showerheads suffer from poor spray delivery along with more pronounced temperature drops compared with the other showerheads. Showerhead 1 suffers from asymmetrical and uneven spray distribution and a small area of high spray intensity despite a similar temperature drop compared with the higher rated showerheads. The better-rated showerheads, in contrast, have well-defined central spray areas of high spray intensity, greater symmetry, and evenness of flow distribution. The temperature variations follow the pattern of the spray volume distribution indicating a close link between the spray volume distribution and thermal losses. A drop of about 2 °C between adjacent zones was found, indicating significant heat transfer from the spray to the surrounding air. This pronounced heat transfer was found to be related to the low spray intensity.

Functionality mismatch, i.e., the desired functionalities and delivered performance, can occur and affect context in which the product is used (Wever et al. 2008) and can result in what can be referred to as unwanted or anticipated side effects (Rooden and Kanis 2000). The user feedback on how their decisions for choosing a showerhead are made, and their perceived and actual shower duration and water use was evaluated. It was found that the participants, when compared with the eco-performance of the product or the need to conserve water, consider the performance factors of the showerheads to be important. The implications are that functionality matching as judged by the user, against his/her goal with the product, remains important for the water use efficiency of products such as showerheads.

Water scarcity is one of those challenges, finding viable solutions necessitates drawing on every tool in the box, and harnessing the potential of underutilized, yet effective, approaches (Lede and Meleady 2019). However, methodology/finding disputes occur in literature where ‘in-home’ user studies do not find attitudes and social norms effective in changing behaviour whilst studies conducted in artificial environments, e.g., laboratories, or in silos, e.g., surveys and interviews have found the opposite (Parker et al*.*^2018^). Despite the limited sample size, particular attention was paid to the research and methodological design of the study as supported by existing literature on studies that focuses on depth rather than breadth of application and findings. The correlations between laboratory tests and user feedback extended the works of Alkhaddar et al. 2007 and Okamoto et al., 2015a and b. Therefore, this study also makes methodological contributions by applying theoretical and lab-based findings to real-world settings to improve the reliability of deductions (after: Kahan and Carpenter 2017; Johnstone and Hooper 2016).

## Conclusion

Domestic water savings can be increased through economic incentives (e.g., water pricing), technical improvements (e.g., water-saving household appliances), or policy instruments and regulation (Koop et al. 2019). Key knowledge gaps were identified in the relationship between the technological performance of water efficiency products, showerheads in particular, and the extent to which these parameters affects user satisfaction, and therefore water-efficient behaviours and practices. This research aimed to fill this knowledge gap by defining, in addition to flow rate, the other indicators that would help define the water use efficiency of water-efficient products. It evaluated the technical efficacy of water-efficient showerheads as a determinant for its social use impact, i.e., efficiency in-use. To achieve this aim, a socio-technical approach was proposed; laboratory studies were conducted, and the findings correlated with user feedback provided during in-home trials of the same showerheads. The justification was that studies such as this contribute to the better understanding of sustainability mismatch in water-efficient products against user preferences and use behaviours. Thus, the combined laboratory experiments, in-home use, and user feedback made it possible to determine the interdependency among the socio-technical factors that inform the purchase and use of water efficiency products thereby helping to improve the design and in-use efficiency of the showerhead.

This study found that the thermal characteristics of the spray may have an influence on the user’s showering experience, but the spray volume distribution has a dominant effect. It also found good merit for including the spray intensity (pressure), distribution, and degree of heat loss, in addition to the discharge rate, as part of the performance and efficiency considerations of showerheads. Therefore, it is recommended, subject to further studies, that these parameters are included in water labels in addition to the flow rate. They can be included simply and diagrammatically to enable intuitive decision making by purchasers and users.

The findings address the lack of awareness that still exists amongst policy makers, designers, planners, engineers, and consumers of the potential for reducing water demand through water-saving devices in conjunction with current and future changes in water use behaviour (Zadeh et al. 2014). They are significant and novel, in that they show that both volumetric and temperature distributions of the spray play an important part in deciding the overall experiential performance of such products from the users’ perspective. This means that the design of water-efficient showerheads, in addition to delivering water discharge savings, should avoid poor spray distribution, intensity, and heat retention. The recent findings by Carbon Brief (2018) found that small but cumulative efficiencies, for instance from the use of energy saving lightbulbs, can have significant impact in reducing the demand for and therefore the carbon impact of energy generation. Therefore, the knowledge derived from studies such as this cannot be under-estimated as it can improve the design, uptake, and efficient use of water-efficient products, thereby cumulatively improving water demand and reducing the carbon/climate impact of water abstraction, treatment, distribution, wastewater recovery, and discharge back to the natural environment.

## Figures and Tables

**Table 6:**
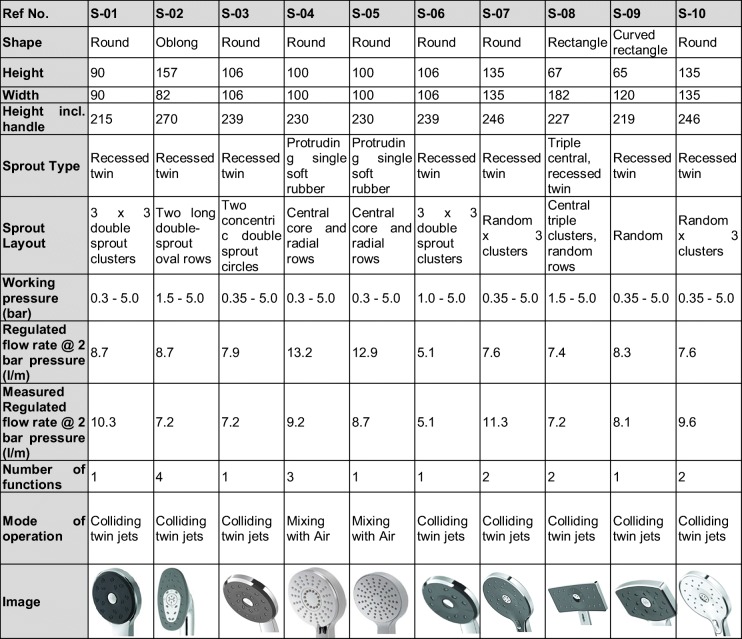
Characteristics summary of the showerheads in the sample

## Appendix

**Table 7:** Mean user feedback (beginning of trial week) per gender

Metrics	Coded feedback (pre and post user trial)	S-1		S-2		S-3		S-4		S-5		S-6		S-7		S-8		S-9		S-10	
		Female	Male	Female	Male	Female	Male	Female	Male	Female	Male	Female	Male	Female	Male	Female	Male	Female	Male	Female	Male
Consistency of flow	Pre_Consistentflow	3.33	4.33	4.17	4.4	3.5	4.4	4	4.4	4	4.63	3	3.6	4.5	4.25	4.5	3.83	4.5	3.5	4.5	4.5
Consistency of pressure	Pre_Consistentpressure	3.33	4.33	4.17	4	3.33	4.4	4	4.6	4	4.63	2.75	3.6	4.5	4.25	4.5	4	4.5	3.5	4.5	4.5
Pressure (force impact) on body	Pre_Pleasantpressure	3	2.83	3.83	3.6	3.25	3.2	3.8	4	3.8	3.5	2.75	3.4	4.5	3.75	4	3.17	4.17	2.75	4	4.13
Feel of the spray	Pre_Spraypressure	3.33	2.83	4	3.6	3.25	3.4	3.8	4.2	3.6	3.75	2.75	3.6	3.75	3.75	3.83	3.5	4.33	3.25	4.25	4.13
Coverage of the spray	Pre_Spraycoverage	3.67	2.17	3.67	3	3.5	3.6	4	4.4	3.6	3.63	3	3.6	3.5	3.75	2.83	3.83	3.67	2.5	4.5	3.75
Acoustics (pleasant and soothing sound)	Pre_Sound	3	3.67	4.17	3.8	3.25	4.4	3.8	4	3.2	3.5	3.5	3.6	4	3.75	4	3.67	4.17	3.75	4.25	4.38
Consistency of temperature	Pre_Temperature	3.67	4.17	4.33	4.8	3.5	4.2	3.8	4.2	3.8	4.25	3.25	4	4	4.25	4.17	3.5	4.5	3.75	4.5	4.13
Had to change shower habit to suit showerhead	Pre_Changehabit	3	2.67	2.5	2.2	2.5	1.6	2.2	1.8	3	2.75	4	2.8	2.5	3.25	3.33	2.17	2.17	2	2.75	2.63
Had to change body positioning in the shower	Pre_Changeposition	3	2.83	3.17	3.2	2.5	2.6	2	2.2	3	3.63	3.5	2.6	3.25	2.75	3.33	2.67	2.67	2	2.75	2.88
Ease of Use	Pre_Ease	4	4.33	4.14	4.4	3.25	4.6	4.2	4.4	4	4.38	2.75	4	4.25	4.25	3.83	4.17	4.33	4	4.75	4.38
Enjoyment during use	Pre_Enjoyableuse	3	2.83	4	3.2	3.25	3.6	3.8	3.8	3.8	3.88	2.75	3.8	4	4	3.5	3.83	4.17	3.25	3.75	4.13
Look (design)	Pre_Look	4.33	3.17	3.43	2.6	3.25	3.4	4.6	3.4	4	3.5	4	3	4.75	4	4.33	3.67	4.33	2.75	4.5	4.25
Effort to clean e.g. hair	Pre_Loweffortclean	3	2.5	2.5	2	2.5	2.2	3	2.4	2.8	2.5	2.25	3	3.25	2.25	3	3	2.83	2.5	2.75	2.63
Showered more than usual	Pre_Morefrequse	2.67	2.67	2.5	2.2	2.75	3	2.4	2.4	2.4	2	2.75	1.6	3.25	3.5	2.5	2.67	3.17	2.25	2.25	2.5
Physical feel (handling)	Pre_Physicalfeel	3	2.67	3.5	2.6	3	3	3.6	3.6	3.4	2.88	3.5	2.4	3.5	4	3.67	3.33	4	2.25	4	3.88
Quickness (ease) to install, stay as installed	Pre_Quicktoinstall	4.67	4.67	4.67	4.4	4	4.2	4.6	4.6	4.2	4.5	4.75	4	4.5	5	4.67	4.67	4.5	4.5	4.75	4.75
Performs as expected	Pre_Worksasexpects	4	3.33	3.83	3.2	3.5	4.2	4	4	3.6	3.75	2.5	4	4	3.5	3.33	3.83	4.33	3.25	4.5	3.75
Continuity of use	Pre_Continueuse	2.67	2.67	3.33	3	2.25	3.6	3.6	4.2	3.4	3.13	2.25	3.4	3.75	3.5	2.67	3.83	3	2.5	3.5	3.5
Willingness to buy	Pre_Happytobuy	2.67	2.33	3	3	2.5	3.4	3.8	4.2	2.6	3.13	2	3	3.75	3.5	2.5	3.5	3	1.5	3.5	3.5
Meets all expectations	Pre_Meetsexpectats	2.67	2.17	3.33	2.4	3	3	3.6	4	3.4	2.88	2.5	3	4	3.5	2.83	3.33	3.67	2.5	3.75	3.5
Preference compared to pre-study showerhead	Pre_Preferencecompare	2.33	2.33	2	2.4	1.5	3.2	3	3.4	3.2	2.88	2.5	3.6	3.25	3	2.83	3.67	3.17	1.75	2.75	2.88
Likelihood to keep	Pre_Willkeep	2.67	2.17	2.83	2.6	1.75	3	3.4	4.2	3	3	1.5	3.2	3.75	3	2.17	3.5	2.67	1.75	3.5	3.38
Personal preference for this showerhead compared to the others	Pre_Worksforme	3	2.67	3	2.4	3	3.4	3.6	4.2	3	3.38	2.25	3.2	4.25	3.5	2.33	3.83	3.17	2	3.5	3.38

**Table 8:** Mean user feedback (end of trial week) per gender

Metrics	Coded feedback (pre and post user trial)	S-1		S-2		S-3		S-4		S-5		S-6		S-7		S-8		S-9		S-10	
		Female	Male	Female	Male	Female	Male	Female	Male	Female	Male	Female	Male	Female	Male	Female	Male	Female	Male	Female	Male
Consistency of flow	Post_Consistentflow	3.33	4.33	4.43	4.4	3.25	4.75	4	4.4	4	4.67	3	3.8	4.25	4	4.33	3.83	4.33	3.5	4.25	4.5
Consistency of pressure	Post_Consistentpressure	3.33	4.33	4.43	4.6	3.25	4.5	4	4.2	4	4.67	3	3.8	4.25	4.25	4.33	3.83	4.33	3.5	4.25	4.5
Pressure (force impact) on body	Post_Pleasantpressure	3	3	4	3.6	3.25	3	3.8	4	3.6	4	2.5	3.2	4.25	3.5	4	3.17	3.67	3.25	3.75	4.25
Feel of the spray	Post_Spraypressure	3	2.67	3.86	3.6	3	3	3.8	4.4	3.6	3.17	2.75	3.4	4	3.5	4.17	3.17	4	3.25	4	4.38
Coverage of the spray	Post_Spraycoverage	3.33	2.17	3.14	3.6	3.75	3.25	4	4.4	3.4	3.83	2.75	3.4	4	4.25	3.33	3.33	3.67	3	4.25	3.71
Acoustics (pleasant and soothing sound)	Post_Sound	3	3.33	4.14	3.6	3.25	4.25	3.6	3.6	3.4	4	2.75	3.6	3.75	3.75	4.17	3.5	4	3.5	4.25	4.13
Consistency of temperature	Post_Temperature	3.67	4	4.29	4.4	3.75	3.75	4	4.4	4	4.17	2.75	4	3.75	4.25	4.33	3.33	4.33	3.75	4.5	4.25
Had to change shower habit to suit showerhead	Post_Changehabit	3.67	2.83	3.14	2	2.5	1.75	2.2	1.8	2.8	3.67	3.75	3.6	2.67	3.25	3.83	2.17	2.83	2	2.5	2.5
Had to change body positioning in the shower	Post_Changeposition	3.33	2.83	3.14	3.2	2.75	2.75	2	2	2.8	3.33	3.75	2.6	3.67	3	3.83	2.67	2.5	1.75	2.75	3.13
Ease of Use	Post_Ease	2.67	4.17	4.29	4.6	4	4.75	4.2	4.6	4.2	4.83	3	3.8	4.75	4.5	3.5	4.17	4.67	4	4.75	4.25
Enjoyment during use	Post_Enjoyableuse	3	2.67	3.86	3.4	2.75	3.75	4	4.2	3.2	2.83	2.75	3.2	4.5	3.75	3.67	3.83	3.83	3.5	3.75	4
Look (design)	Post_Look	4	3	3.71	3.2	3	3.75	4.2	4	4	3.33	4.5	3.2	4.5	4.25	4.33	3.67	4.5	2.25	4.5	4.25
Effort to clean e.g. hair	Post_Loweffortclean	2	2.17	1.71	2	2.5	2.25	3.4	2.4	3.2	2.33	2.5	3	3	2.5	3	3.5	2.67	2	2.75	2.88
Showered more than usual	Post_Morefrequse	2.67	2.67	2.14	2.4	1.5	2.75	2.4	2.2	2.6	2.5	2.5	1.6	3	3.25	2.83	2.67	3	2	2	1.88
Physical feel (handling)	Post_Physicalfeel	3.33	2.67	3.43	2.8	2.75	3.25	3.6	3	3.2	2.83	2.5	2.6	4.25	4.25	3.67	3.5	3.83	1.75	3.75	3.88
Quickness (ease) to install, stay as installed	Post_Quicktoinstall	4.67	4.5	4.57	4.8	4.25	4.5	4.6	4.8	4.8	4.33	4.5	4.4	4.75	4.75	5	4.67	4.67	4.5	4.75	4.5
Performs as expected	Post_Worksasexpects	2	3.5	3.57	3	3.5	4.25	4	3.6	3.2	3.5	2.25	3.8	4	3.25	3.67	3.83	4	3.25	4	3.88
Continuity of use	Post_Continueuse	2	2.33	2.86	2.4	2.25	3.25	3.4	3.8	2.6	2.67	2.25	2.8	4	3.75	3	3.5	3.17	2.25	3.75	3.75
Willingness to buy	Post_Happytobuy	2.33	2.33	2.71	2.6	2.5	3	3.6	4	2.2	2.5	2.25	2.6	4	3.5	2.67	3.5	3.17	2.25	3.5	3.5
Meets all expectations	Post_Meetsexpectats	2.33	2.33	3.71	2.6	2.75	2.75	3.6	3.8	3	2.33	2.25	3.2	4	3.5	3.33	3	3.83	2.25	4	3.5
Preference compared to pre-study showerhead	Post_Preferencecompare	2	2.33	2.29	2.6	2	3	3	3.6	2.8	2.83	2.25	3.4	3.67	3	3.17	3.83	2.33	2.5	3	3.13
Likelihood to keep	Post_Willkeep	1.33	2	2.29	2.2	2.25	3	3.2	3.8	2.2	2.5	1.75	2.2	4	3.25	2.83	3.5	2.83	2	3.5	3.5
Personal preference for this showerhead compared to the others	Post_Worksforme	2	2.5	3.14	3	2.5	3.5	3.8	4	2.8	2.5	2.25	2.8	3.75	3.5	2.83	3.5	3.5	2.75	3.75	3.5
